# Collaborative modeling of an implementation strategy: a case study to integrate health promotion in primary and community care

**DOI:** 10.1186/s13104-017-3040-8

**Published:** 2017-12-06

**Authors:** Gonzalo Grandes, Alvaro Sanchez, Josep M. Cortada, Haizea Pombo, Catalina Martinez, Laura Balagué, Mary Helen Corrales, Enrique de la Peña, Justo Mugica, Esther Gorostiza, on behalf of the PVS group, Gonzalo Grandes, Álvaro Sánchez, Haizea Pombo, Josep M Cortada, Catalina Martínez, Paola Bully, Aitor Sanz-Guinea, Carlos Sola, Martín Begoña, Susana Iglesias, Maite Cuadrado, Nuria González, Teresa Garmendia, Mª Luz Jáuregui, Amaia Hernando, Jesús Larrañaga, Maribel Romo, Pilar Isla, Enrique Maíz, Cristina Domingo, Carmen Esparta, Mª Luz Marqués, Encarnación San Emeterio, Antón Elorriaga, Justo Múgica, María Pilar Alberdi, Mª Ángeles Arrondo, Amaia Azkoitia, Xabier Epaizabal, Mª Aranzazu Echeverria, Mª Esperanza García, Mª Ángeles García, María Erkuden Imaz, Mª Antonia Iparraguirre, Mª Isabel Irizar, Mª Rosario Larrea, Mª Dolores López, Petra Pacheco, María Yolanda Porres, Begoña San Juan, Mª Aranzazu Suquia, Mª Teresa Arrospide, Carolina Díez, Miren Arantxa Igartua, Oihana Jauregui, Alazne Saizar, Mª Jose Tilves, Mª Lourdes Etxeberria, María Aurora Valdivielso, Xabier Mugica, Mª Mercedes Lasagar, Coro Zabaleta, Mª Isabel Urcelay, Mary Helen Corrales, Mª Ángeles Crespo, Javier José María Jesús de Ordozgoiti, María Iciar Elguezabal, Susana Esteban, Catalina Frau, Laura Gallo, Inés Yolanda Martín, Nerea Ordorika, José Ramón Pérez, Mª Begoña Relloso, María Soledad Sangroniz, María Iluminada Santos, Patxi Xabier Iturbe, Esther Gorostiza, Mª Esther Azpitarte, Bixente Barrutia, Amaia Bengoa, Francisco José Miguel, Ana Isabel Etxebarria, Mª Belén García, Mª Jose Ibars, Mª Jose Lasa, Mª Carmen Martínez, Maura Pernudo, Lourdes Oribe, Mª Dolores Ustarroz, Valentina Camino, Leire Corpión, Leire Ortuondo, Mª Carmen López, Rosana Abraldes, Eneko Ibarruri, Javi Alonso, Enrique de la Peña, Mª Carmen Artola, Teresa Casado, Jesús García, Mª Paz Sánchez, Luisa Santos, María Lanzarote, Concha Castells, Francisco Cirarda, Henar Ortuondo, Pilar Manrique, Ines Urieta, Amaia Ajuria, Ricardo Ortega, Jesús Torcal, Mª Sol Ariestaleanizbeaskoa, Verónica Arce, Álvaro Sánchez, Gonzalo Grandes, Bittor Rodríguez, Pilar Amiano, Esther Gorostiza, Enrique de la Peña, Álvaro Sánchez, Gonzalo Grandes, Esther Azpitarte, Mary H. Corrales, Josep Cortada, Álvaro Sánchez, Gonzalo Grandes, Juan Manuel Elosegui, Iñaki Korta, Ainhoa Irastorza, Leire Makibar, Jon Alkaiaga, Karmele Alkaiaga, Iñaki Aldamiz, Virginia Zelaia, Adela Etxeberria, Itziar Basurto, Gonzalo Casado, Maite Martínez, Alberto Díez, Javier Rojo, Sara Garteiz, Aitziber Artabe, Ainhoa Parra, Marcelo Borja, Mª Carmen Jiménez, Gorka Carro, Bernardo Valdivielso, Janire Kasuso, Esther Martin, Paulino Parra, Loinaz Albizu, Marivi Cuartango, Unai Atxa, Jesús Miguel Enríquez, Rosario Acebal, Javier Ancel, José Luis Navarro, Inmaculada Zapardiez, Nerea Lejarzaburu, Edurne Madariaga, Eugenia Peral, Pablo Mas, Juan Mayor, Joseph Reverte, Bernard Mandaluniz, Roberto Nuño, Josu Llano, Enrique Gutiérrez, Maribel Cifuentes, Gonzalo Bacigalupe, Marie-Pierre Gagnon

**Affiliations:** 1 Primary Care Research Unit of Bizkaia, Basque Healthcare Service-Osakidetza, BioCruces Health Research Institute, Luis Power 18, 48014 Bilbao, Spain; 2 Deusto Primary Health Care Center, Bilbao-Basurto Integrated Care Organization-Osakidetza, BioCruces Health Research Institute, Luis Power 18, 48014 Bilbao, Spain; 3 Iztieta Primary Care Center, Donostialdea Integrated Care Organization-Osakidetza, Avenida de Lezo, s/n, 20100 Renteria, Spain; 4 La Merced Primary Health Care Center, Bilbao-Basurto Integrated Care Organization-Osakidetza, Luis Iraurrizaga 1, 48003 Bilbao, Spain; 5 Sondika Primary Health Care Center, Uribe Integrated Care Organization-Osakidetza, Lehendakari Agirre 41, 48160 Sondika, Spain; 6 Beasain Primary Health Care Center, Goieri-Alto Urola Integrated Care Organization-Osakidetza, Bernedo s/n, 20200 Beasain, Spain; 7 Matiena Primary Health Care Center, Barrualde-Galdakao Integrated Care Organization-Osakidetza, BioCruces Health Research Institute, Trañabarren 13-Bajo, 48220 Abadiño, Spain

**Keywords:** Primary health care, Health promotion, Health education, Preventive care, Implementation strategies, Implementation research, Community of practice, Participatory action research, Learning community, Health information technologies

## Abstract

**Background:**

Evidence-based interventions are more likely to be adopted if practitioners collaborate with researchers to develop an implementation strategy. This paper describes the steps to plan and execute a strategy, including the development of structure and supports needed for implementing proven health promotion interventions in primary and community care.

**Results:**

Between 10 and 13 discussion and consensus sessions were performed in four highly-motivated primary health care centers involving 80% of the primary care staff and 21 community-based organizations. All four centers chose to address physical activity, diet, and smoking. They selected the 5 A’s evidence-based clinical intervention to be adapted to the context of the health centers. The planned implementation strategy worked at multiple levels: bottom-up primary care organizational change, top-down support from managers, community involvement, and the development of innovative e-health information and communication tools. Shared decision making and practice facilitation were perceived as the most positive aspects of the collaborative modeling process, which took more time than expected, especially the development of the new e-health tools integrated into electronic health records.

**Conclusions:**

Collaborative modeling of an implementation strategy for the integration of health promotion in primary and community care was feasible in motivated centers. However, it was difficult, being hindered by the heavy workload in primary care and generating uncertainty inherent to a bottom-up decision making processes. Lessons from this experience could be useful in diverse settings and for other clinical interventions. Two companion papers report the evaluation of its feasibility and assess quantitatively and qualitatively the implementation process.

## Background

Evidence-based interventions are more likely to be taken up if users of these interventions collaborate with researchers in the development of an effective and research-informed implementation strategy, including structure and supports that help these users to change their practice and organization to perform the proven intervention [1–5]. This type of collaborative bottom-up approach is especially necessary when implementation strategies are conceptualized not only as complex procedures but also as social processes, in which professionals take up a specific intervention or innovation if they chose to do so and creatively apply it in their setting, solving competing interests and reaching group consensus on re-design of their care delivery system [5–9]. While few studies appropriately report the details of their implementation strategies [10], there are even fewer describing the process through which these strategies were designed and tailored [11]. The report of the carrying out of this kind of collaborative experience is essential to learn from the process and to inform future refinement and replication.

Our target for improvement was the integration of healthy lifestyle promotion within primary and community healthcare. Health promotion is an excellent example of the need for implementation strategies because of the huge gap between evidence and practice in this area. Despite the sound epidemiological evidence for the impact of individual behavior on population health [12–18], we are failing to progress in the adoption of a healthy lifestyle: less than 10% of the population in developed countries do regular physical activity, follow a balanced diet, do not smoke and do not drink to excess, and the great majority have multiple behavioral risk factors [19, 20]. In addition, the current economic crisis underlines the critical role of the prevention of chronic diseases associated with these behaviors in the sustainability of healthcare systems [21] and primary care practitioners are in the best position within these systems to promote healthy behaviors among the population due to their accessibility and role in providing continuity of care [22].

Nevertheless, despite the availability of effective evidence-based interventions, healthy lifestyle promotion is far from being integrated into routine clinical practice in primary care [23–25]. For example, during the last decade our own research group has contributed with evidence-based clinical interventions for health promotion in routine primary care based on clinical trials [26–28]. Nevertheless, we have to recognize that after these trials finished participating clinicians stopped delivering the interventions [29]. The main reasons which explain this lack of sustainable integration are the inherent difficulties and complexities of changing, on the one hand, people’s lifestyles [23, 24, 30, 31], and on the other, clinical practices and the organization of primary care services [2, 23, 32, 33]. As a consequence, as in many other examples, valuable innovative initiatives fail due to implementation weaknesses [34, 35].

**Fig. 1:**
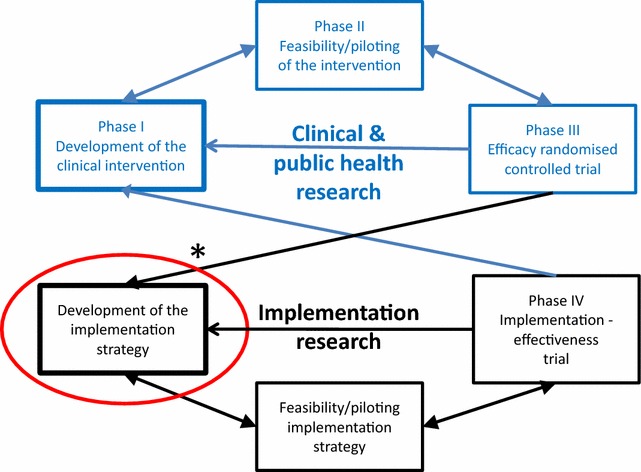
Extended framework to include implementation research in the process of developing and evaluating complex interventions. Modified from Pinnock et al. [37]. Reproduction authorized by the Editors

In accordance with the complexity of developing a targeted implementation strategy, we worked step-by-step following the UK Medical Research Council guidance for the development and evaluation of complex interventions [36]. In this paper, we describe the first step of this process, which is better visualized in relation to the extension of this guidance proposed by Pinnock et al. for phase IV implementation studies [37] (see Fig. 1). As previous preparatory work, an expert panel analyzed the causes of the implementation gap and identified roadblocks to change [23, 29]. In brief, they recommended a process of mutual adaptation of evidence-based interventions to the specific context of the primary health care (PHC) centers and, in turn, redesigning the practices and organization of these centers with the active participation of the healthcare practitioners and managers of these services, researchers and community members. They also recommended adopting the Chronic Care Model as a validated general framework to guide the redesign of primary care delivery necessary to integrate health promotion into routine practice [38–40]. Therefore, possible actions to be included in the strategy were considered at different levels: self-management support, delivery system redesign, decision support, e-health tools integrated into the clinical information systems, community resources, and health system organization.

This paper describes the process of how to engage primary care staff and members of the community to reorganize primary care delivery system to optimize physical activity, healthy diet and smoking cessation interventions in primary and community healthcare. This is the companion article of two recently published in a series documenting the development and subsequent piloting of the Prescribe Healthy Life implementation strategy (PVS—from the Spanish ‘*Prescribe Vida Saludable*’-) (Fig. 1) [41, 42].

## Methods

Action research principles were used to collaboratively model a multi-component implementation strategy for health promotion interventions [1–4]. This was a bottom-up process of dialogue, discussion and consensus among a multi-professional primary care team and community members for shared decision making on actions to be included in the implementation strategy. The study protocol has been published previously [43]. Briefly, we refer to this method as collaborative modeling, defining this as the process of redesigning the primary healthcare delivery system with a dual purpose: on the one hand, adapting available evidence-based health promotion interventions to the context of each of the collaborating PHC centers, and on the other, reaching a consensus among professionals and community members on reorganizing the practice delivery system, creating a multi-professional workforce, defining new professional roles, and redistributing tasks and workflows. Intervention mapping was used as a guide to schedule structured discussion/consensus meetings and the Institute of Medicine Plan-Do-Study-Act improvement cycles were carried out [44, 45]. The study protocol was approved by the Primary Care Research Committee of the Basque Health Service and by the Basque Country Clinical Research Ethics Committee (Ref: 06/2009).

### Setting and participants

Four public community PHC centers were selected for convenience by managers of the district primary care organizations on the basis of their especial motivation favorable to health promotion [42]. For a candidate center to be included, individual written commitment to the project was required by a majority of the staff within each of the professional categories (administrative and clerical staff, nurses, family physicians, pediatricians, and others), after an informative session in the center explaining the objectives of the project and the work plan. Primary care professionals of these centers, managers of the Basque Health Service, community partners, and researchers were engaged to model the implementation strategy. A local champion was identified in each of the collaborating centers and on-site supportive practice facilitation was provided by the research team.

The Basque Health Service (Osakidetza) provides universal coverage free at the point of delivery funded through regional general taxation. In Spain, primary care services are almost exclusively delivered in publically-owned centers. Each citizen is registered on the list of one family physician or pediatrician, and these clinicians work in PHC teams including nurses and administrative personnel. They provide comprehensive primary care with easy accessibility for residents in a defined geographical area (70% of the population visiting their family physician at least once a year). Healthcare staff have a civil-servant like employment status and they are paid a fixed salary with small capitation payments for physicians.

### Modeling the implementation strategy

Table 1 summarizes the stages, planning and quality improvement techniques used for modeling the implementation strategy. This process was organized through discussion and consensus meetings to assess needs, prioritize areas for improvement and select common goals; provide education on evidence-based health promotion interventions and selection by PHC center staff and community members of the most appropriate clinical interventions to be implemented; make consensus on how to redesign workflows and redistribute tasks; and then brief piloting; followed by training, auditing and feedback (see Table 1). The research team acted as practice facilitators for this modeling process, including organizing and summarizing meetings, providing selected documentation and periodical activity reports. A local coordinator was selected at each center that was the liaison with the research team and leaded the process at the local level.

**Table 1:** Steps in the collaborative modeling of the PVS implementation strategy under a participatory action research framework involving primary care staff and community members supported by external facilitation provided by the research team

Implementation goals	Contents and activities	Techniques of consensus, planning, quality improvement and evaluation
1st—descriptive stage (three or four 90–120 min sessions)		
To obtain the *commitment* of the majority of the professionals to a common health promotion goal, after prioritizing which behaviors and groups to target	Presentation of PVS objectives and plan Assessment of attitudes, perceived practice and organizational climate in the primary health care center Gathering of information on general and local epidemiology of unhealthy behaviors	Strategic evaluation of healthy lifestyle promotion practice (audit and feedback) Individual identification of areas for improvement (gaps and needs assessment) Prioritization and consensus (nominal group)
2nd—creative stage (three 90–120 min sessions)		
To acquire *competence* in planning the preliminary intervention program: specify objectives and actions, and identify agents and resources involved	Analysis of determinants of behavior Review of evidence-based interventions Tailoring of interventions to the actual context of the center Redesign of workflows and staff roles	Educational sessions on theoretical models and health promotion interventions Group discussion—consensus and planning Specific objectives (behavior determinants) Mapping of actions and interventions Redistribution of tasks and responsibilities: what, who, how, when and where
3rd—piloting stage (four to six 90–120 min sessions)		
To achieve active *cooperation* among the multidisciplinary team within the center and with community agents, optimization of intervention components and their sustainable integration	Practical exercise of implementing intervention actions in real-world conditions, for the identification of feasible strategies Monitoring of performance measures Standardization of preliminary program on the basis of feasibility	Brief Plan-Do-Study-Act cycles for piloting Audit and feedback in group sessions Learning sessions to identify readjustments Strategic evaluation of the center’s capability to address the planned programs and availability of resources necessary for their implementation (SWOT matrix)

The main contribution at the management level was to ensure the availability of new information and communication technology tools, intervention materials, and other resources necessary to facilitate the organizational change at the level of the primary care system. Further, management were required to set aside time 1 day a week for the local champion of the program in each center to support and supervise implementation at local level, and on average 2 h a month for the discussion and consensus meetings within working hours, to allow participation of the entire primary care team, covering clinical and administrative tasks with additional staff. District primary care authorities also lent institutional support to the project, which helped to initiate coordination with community organizations.

At the community level, PHC center staff were asked to identify potential partners and resources in their primary care catchment area. The research team contacted these community agents by letter, informed them of the objectives of the project, and invited them to collaborate in the subsequent sessions of the collaborative modeling. Public health practitioners from the Basque Department of Health and Consumer Affairs contributed to link community organizations and the PHC centers.

### Data and analyses

First, we describe actual engagement of professionals and community in the process of modeling the implementation strategy. To this end, we documented their participation in each of the steps of the implementation strategy by asking participants to sign a register at each event, keeping signed registers of attendance at meetings, and writing summary reports of each of the meeting listed in Table 1. Second, based on the abovementioned documentation, we outline the final implementation strategy designed in terms of clinical actions to be performed, distribution of tasks between participants, definition of new roles assigned to each participant, and the new organization of the health promotion delivery system. Third, we describe the experience of professionals involved. At the end of all the collaborative modeling sessions listed in Table 1, a final meeting was organized for qualitative evaluation. The nominal group technique was used to explore the opinions of primary care professionals about their experience in the process of collaborative modeling of the PVS program [46]. As a preparatory part of the nominal group technique participants were surveyed about the positive and negative aspects of the collaborative process, the implementation climate, facilitators and barriers related to the feasibility of the implementation strategy designed, and the relationship between researchers and PHC center staff. The results of the survey were summarized and reported back to the group followed by a 90-min open-group discussion session to prioritize the most relevant aspects of the process of modeling the PVS implementation strategy.

## Results

**Fig. 2:**
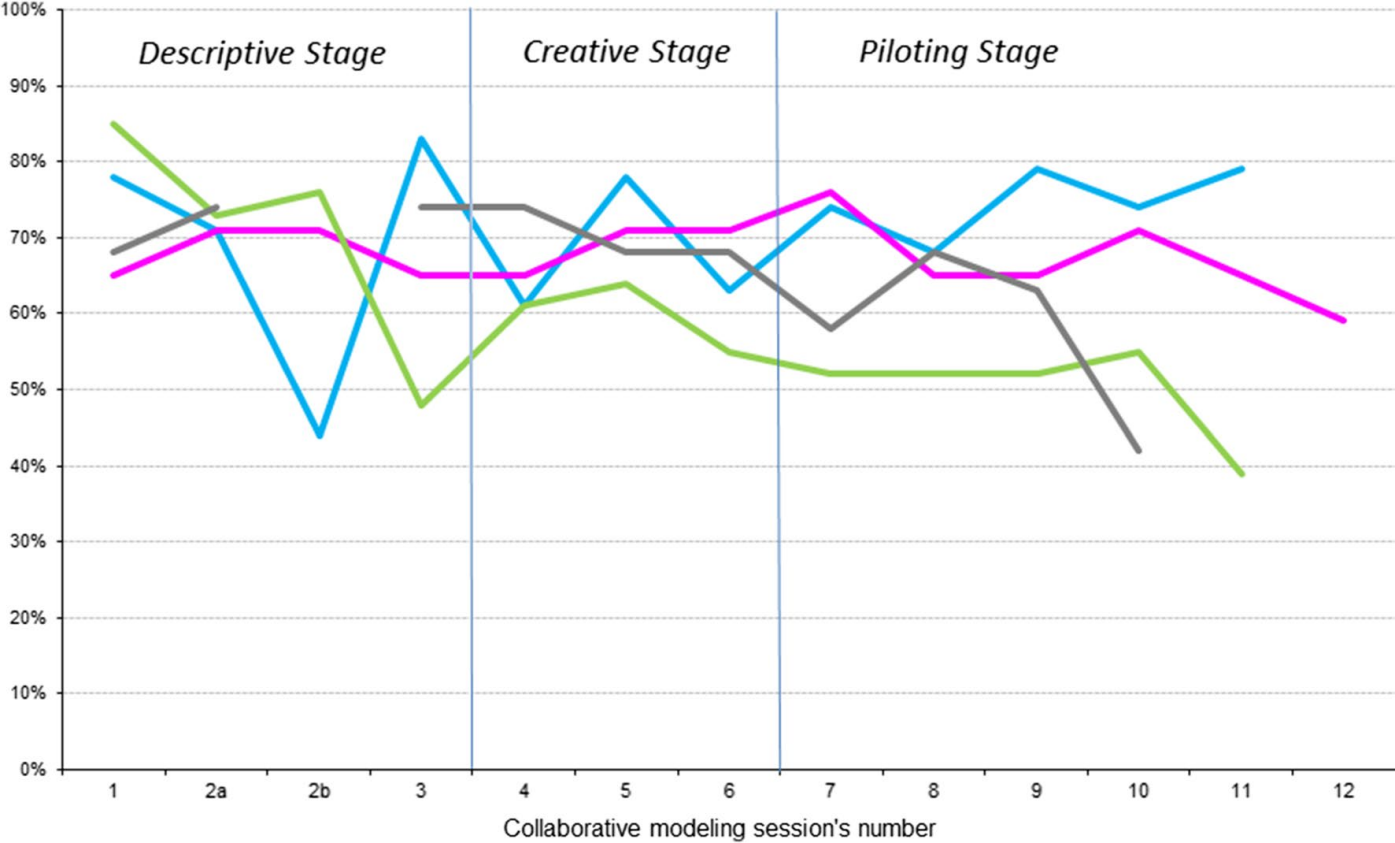
Percentage of professionals who participated in each of the collaborative modeling sessions out of the total number of professionals of the primary care center

The same sequence of meetings was performed at each PHC center for collaborative modeling of the PVS implementation strategy. It required different numbers and lengths of discussion and consensus meetings at each center, ranging from 10 to 13 structured sessions lasting between 90 and 120 min. Active engagement of 71 (80%) of the 89 staff working in the four PHC centers was achieved. The highest proportion of participation was observed among family physicians (n = 24, 92% of the 26 working in the four PHC centers), followed by reception staff (n = 18, 86%), nurses (n = 23, 74%), pediatricians (n = 3, 50%), and midwives (n = 2, 50%), while the only dentist also participated. The staff participation remained above 50% in all of the discussion and consensus sessions in one center, in all but one session in two other centers, and in all but two sessions in the fourth center (Fig. 2).

**Fig. 3:**
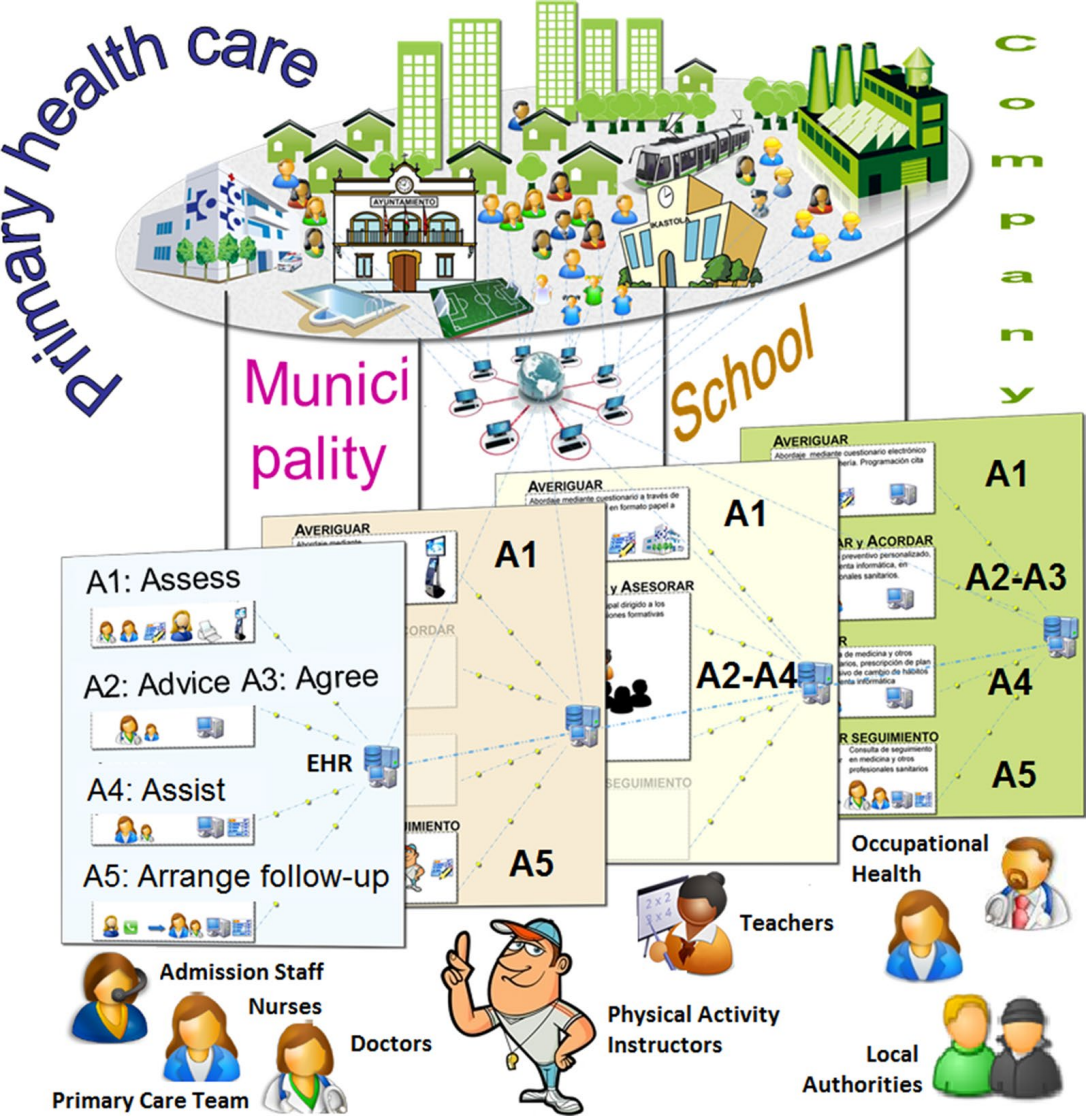
Structure and actions of the new PVS health promotion strategy

During the creative phase (Table 1), participants selected four theoretical models of behavior change as a basis for their programs, among the models most frequently used for health promotion [47]: the health belief model, the theory of planned behavior, the transtheoretical model and the social-cognitive model. The 5 A’s (Assess, Advise, Agree, Assist, and Arrange follow-up) behavioral counseling intervention was identified in all four PHC centers as the most effective and feasible evidence-based clinical intervention for the objectives set [48]. Specific tasks, goals and actions were distributed among the primary care staff as shown in Table 2 and Fig. 3. Some examples of the planned distribution of clinical intervention tasks between participants are the following. The “Assess” step was performed by receptionists before patients were seen by physicians, outside the center by school teachers, by company occupational health departments, or by individuals themselves through the Internet. The “Advice” and “Agree” steps were mainly delivered by family physicians or company doctors. The “Assist” step was mainly performed by nurses. All participants inside and outside the centers were involved in the follow-up process with particular involvement of receptionists and nurses (Table 2).

**Table 2:** Targets, actions and agents of the new PVS programs to promote physical activity, healthy diet and smoking cessation in primary and community care

Specific behavioral-cognitive objectives	Intervention actions	Who and How
Identify unhealthy lifestyle behavior and at-risk population Identify attitudes and intention to change to healthier lifestyles	A1 assess: assessment of healthy lifestyle behavior and intention to change	Population self-evaluation through web-based questionnaire linked to electronic health record PVS questionnaires provided to eligible population at the health care center or through community resources (schools, sport facilities, collaborating companies, etc.) Data entry into the electronic health record by scanning by administrative staff or manually by clinicians
Increase perceptions of severity of risks and vulnerability associated with unhealthy lifestyles Strengthen beliefs and knowledge regarding healthy lifestyles and their positive consequences Increase intention to change behavior Strengthen positive beliefs and knowledge regarding healthy lifestyle at the community level	A2 advise: personalized verbal advice centered on the benefits and risks of lifestyle choices A3 agree: assessment of intention to change behavior and agreement of general change goals	Physicians or nurses^a^, guided by PVS software tools included in the clinical information system in routine or scheduled appointments A four-page pamphlet summarizing the abovementioned information on benefits, risks, motivation, and help offered by health care professionals Communication and diffusion of information strategies such as informal talks given by health care professionals in community settings
Enhance self-efficacy perception for behavior change Decrease perception of barriers to behavior change Strengthen coping skills and self-management abilities to facilitate behavior change Improve knowledge regarding community resources to facilitate and support behavior change and prevent relapse	A4 assist: reinforcement of reasons and intention to change Identification of barriers to and solutions for behavior change Prescription of a behavior change plan through specific goal setting and action planning, including a self-monitoring log	Nurses^b^ assisted by PVS software, which includes tools for action planning, time management, database with contact information for community resources, and health problem-tailored information (evidence-based information on benefits related to a variety of health problems) Provision of a folder containing a brief guide to behavior change with the printed prescription attached
Increase reinforcement related to progress in behavior change and health improvements Strengthen perception of support for behavior change within health care, family and community contexts	A5 arrange follow-up Review of behavior change plan, reinforcement centered on achievements, relapse prevention advice and plan re-design May include referral to community resources	Recall system managed by administrative personnel Nurses^b^ in scheduled appointment assisted by PVS software, which includes tools for review and re-design of behavior change plans and a database with contact information for community resources

^a^In primary health care center, although in some cases collaborating companies may also do this

^b^Mainly nurses in the primary health care center and/or at collaborating companies, in some cases family physicians may also do this

Innovative e-health tools were developed and integrated into the electronic health record (EHR) to guide PHC professionals in the process of assessment and tailored delivery of the clinical intervention for the management of healthy lifestyles (regular physical activity, adequate diet and abstinence from smoking) [41].

At the community level, 30 organizations or institutions were contacted and 21 (70%) agreed to participate in the collaborative modeling process and were actively involved in the identification and prioritization of the health promotion goals of the programs. They also participated in the design of the interventions, as well as in piloting the programs. Of the 21 community participants, nine were local authority departments, six were schools, four were sports facilities and two were manufacturing companies. The community participants mostly contributed to healthy lifestyle assessment (e.g., questionnaires administered in schools), some also participated in the advice and support steps (e.g., physicians and nurses of the occupational health departments of the collaborating companies) and in arranging follow-up actions (e.g., referral to sports facilities). In three of the four neighborhoods, local PVS health promotion councils have been set under the local authority to foster and strengthen linkages between clinical practices and community organizations. The main objectives of these councils are to identify and make information available about resources and facilities for health promotion in the community, to increase communication between organizations, and to identify referral mechanisms between them.

The experiences of participants were mixed, with both positive and negative feelings. Among the positive aspects, PHC center staff agreed that the modeling process enhanced the importance of healthy lifestyle promotion in primary care. They highlighted the engagement of the entire primary care team, including reception staff and community members, in shared decision making and cooperation in a community-based program. In addition, the availability of technological tools integrated in the EHR for supporting the clinical interventions was rated positively. Lastly, they also valued the fact that the discussion and consensus meetings were held within working hours.

As for the negative aspects of the process, the PHC center staff emphasized the heavy workload associated with the new health promotion activities compounded the problem of lack of time in the routine context of primary care. They noted the “awkward” language used in the theoretical educational sessions, and, above all, the feeling of uncertainty inherent in the innovation process: “not knowing where the process will end”. Some of the participants felt that there was a hidden agenda managed by the research team to lead them to some predetermined outcome. Additionally, participants pointed out the inherent difficulty of changing behaviors, the complexity of health promotion interventions, and the difficulty to achieve short-term results.

Participants highlighted the following critical areas for optimization to enhance the feasibility and sustainability of the modeled implementation strategies for future application: (a) at the PHC center level, first of all reorganization of on-demand care to minimize work overload, to improve the coordinated flow of care to avoid extra visits by patients, with coordinated working at all professional levels, to foster communication between different tiers of professionals and to provide sufficient staff resources; (b) concerning the information system, improvement of efficiency and reliability of the information and communication tools and databases integrated into the EHR; (c) at the patient level, innovative ways of motivating patients not ready to change and ensuring continuity of care for those with intention to change behavior to minimize false expectations; and (d) at the community level, improvement of coordination with community resources to align forces and avoid duplication of efforts.

## Discussion

This paper illustrates a real world example of developing an implementation strategy through a collaborative bottom-up process engaging PHC staff, community agents and researchers. The three steps followed in this process (Table 1: descriptive, creative, and piloting stages) pursue three Implementation goals needed to introduce change into an organization: first, commitment to a shared common goal; second, planning competence to tailor evidence-based interventions to the different context of each center; and third, real cooperation among the entire group of participants [49].

All the centers chose the 5 A’s clinical intervention and this is probably due to its simplicity, meaning that less time and training are required than for other interventions, and because of the strong scientific evidence available of its effectiveness in the general population [46]. We used the Chronic Care Model as a framework to guide this effort to redesign a healthcare delivery system with the goal of improving health promotion in primary and community care [38–40]. Our approach to changing and reorganizing clinical practice is consistent with newer frameworks such as the Consolidated Framework for Implementation Research, which considers five major domains that may influence successful implementation of healthcare interventions: intervention characteristics, outer setting, inner setting, characteristics of the individuals involved, and the process of implementation [50]. Such frameworks provide no specific blueprints on how they should be operationalized in practice and researchers trying to design implementation strategies for health promotion interventions need detailed examples such as that provided herein on how they should be used [51].

Cooperation among all the PHC center staff and linkage with community agents are extremely challenging and complex social processes [5–9, 52–54]. Consequently, small steps that make progress in this direction should be considered very important. In our experience, these processes present considerable challenges. Firstly, it is not easy to sustain the commitment of staff to the common goal of integrating health promotion into routine practice, over the course of the long process of modeling and implementation. Secondly, the development of useful and efficient information and communication support tools for addressing healthy lifestyle promotion in routine primary care practice should be accelerated. Thirdly, there is resistance to organizational changes, which are essential for successful cooperation among professionals in the implementation of intervention programs. Fourthly, it would be desirable to prioritize health promotion objectives, to avoid conflicts with multiple other activities in daily practice.

All these difficulties are consistent with findings in previous initiatives for integration of health promotion in primary care [55, 56]. Institutional support from managers of healthcare services, to facilitate and ensure the organization and execution of group dynamics in each center, is essential to address these difficulties. In particular, setting aside time in the agenda of practitioners and provision of substitutes to cover regular duties of all staff to free them to attend are necessary requirements to ensure participation of PHC center staff in discussion/consensus meetings.

Prescribe Healthy Life strategy (from the Spanish ‘Prescribe Vida Saludable’) was greatly influenced by previous programs such as Prescription for Health or STEP-UP, carried out in primary care practice-based research networks in the USA [55, 56]. In turn, factors associated with the successful implementation of PVS are similar to those that arose in those programs, i.e., selection of the 5 A’s intervention strategy, active participation of primary care professionals in the decision making process to adapt the intervention to a specific context, the development of innovative information and communication technologies, and linkage with community resources. The PVS project may serve as an example for other primary care services of how to do this.

The main limitation of this study is the selection of centers by convenience. It would have been desirable to measure the readiness for change of the PHC centers, a necessary condition for quality improvement, and use this information in the selection of participating centers. However, measuring organizational readiness for change is not an easy task [57]. Past performance of the organization, the main selection criteria used in this study, is probably the best predictor of successful improvements [58], along with leadership and coaching by facilitators [2, 34, 59]. The two companion papers by Sánchez et al. and Martinez et al. [submitted] evaluate quantitatively and qualitatively the feasibility/piloting of the implementation strategy (see Fig. 1). In brief, they identify a set of key factors that facilitate or hinder the PVS program implementation, show that it is feasible to improve its uptake in routine clinical practice and that contextual factors conditioned each center’s performance [41, 42].

## Conclusions

This detailed description of the design of the PVS implementation strategy can be used by implementation researchers for planning their implementation research projects and will help readers to understand the two companion papers, which describe quantitative indicators of adoption and implementation, as well as PHC center staff’s qualitative perception of the performance of the described strategy. The development of the strategy has been difficult and complex. Lessons learned will be used to improve the implementation strategy and test it in a future experimental implementation trial we are currently planning.

## Abbreviations

5 A’s: Assess, Advise, Agree, Assist, and Arrange follow-up
PVS: Prescribe Healthy Life strategy (from the Spanish ‘Prescribe Vida Saludable’)
PHC: primary health care
EHR: electronic health record
